# No effect of testosterone on behavior in aged Wistar rats

**DOI:** 10.18632/aging.101096

**Published:** 2016-11-12

**Authors:** Veronika Borbélyová, Emese Domonkos, Janka Bábíčková, Ľubomíra Tóthová, Martin Bosý, Július Hodosy, Peter Celec

**Affiliations:** ^1^ Institute of Molecular Biomedicine, Faculty of Medicine, Comenius University, 811 08 Bratislava, Slovakia; ^2^ Institute of Physiology, Faculty of Medicine, Comenius University, 813 72 Bratislava, Slovakia; ^3^ Institute of Pathophysiology, Faculty of Medicine, Comenius University, 811 08 Bratislava, Slovakia; ^4^ Department of Molecular Biology, Faculty of Natural Sciences, Comenius University, 842 15 Bratislava, Slovakia; ^5^ Biomedical Research Center, Slovak Academy of Sciences, 831 01 Bratislava, Slovakia; ^6^ Department of Animal Physiology and Ethology, Faculty of Natural Sciences, Comenius University, 842 15 Bratislava, Slovakia

**Keywords:** andropause, gender, aging, steroids, brain functions

## Abstract

In men, aging is accompanied by a gradual decline in androgen secretion. Studies suggest beneficial effects of endogenous and exogenous testosterone on affective behavior and cognitive functions. The aim of this study was to describe behavioral and cognitive sex differences and to analyze the effects of long-term androgen deficiency in aged male rats. Thirty-months old rats divided into three groups (males, females and males gonadectomized as young adults) underwent a battery of behavioral tests assessing locomotor activity, anxiety, memory, anhedonia, sociability and depression-like behavior. No major effect of gonadectomy was found in any of the analyzed behavioral measures in male rats. The only consistent sex difference was confirmed in depression-like behavior with longer immobility time observed in males. In an interventional experiment, a single dose of testosterone had no effect on gonadectomized male and female rats in the forced swim test. In contrast to previous studies this comprehensive behavioral phenotyping of aged rats revealed no major role of endogenous testosterone. Based on our results long-term hypogonadism does not alter the behavior of aged male rats, neither does acute testosterone treatment. Whether these findings have any consequences on androgen replacement therapy in aged men remains to be elucidated.

## INTRODUCTION

Male and female brains differ in their structure and function [1–3]. In humans and rodents, it has been shown that these differences begin early in development and are mainly caused by the action of sex steroids such as testosterone [4–6]. Brain masculinization in male animals in contrast to females was proved in many behavioral characteristics including affective performance and cognitive function. Previously, gender differences were observed in locomotor activity, [7] anxiety- [8] and depression-like behavior [9], sociability [10] and cognitive function [11]. Sex-specific behavior and its variability are associated with the interindividual as well as intraindividual variability of the endocrine network, especially of the sex hormones.

Aging is associated with a decline in sex steroid hormones. The loss of bioavailable androgens during aging increases the risk of dysfunction in androgen responsive tissues including the brain [12]. It was shown, that cognitive and affective performance and also social behavior decline in aging [13–15]. Whether sex differences in behavior are affected by the age-related androgen depletion is not clear.

In men, the association between androgen depletion and affective or cognitive performance is evident from studies that have analyzed mood, [16] cognitive decline [15] and depression [17, 18] in hypogonadal men. The causality of the relationship between declining testosterone concentrations and behavioral symptoms associated with aging is, however, not firmly established. Rodent models of age-related behavioral changes are used to mimic features of human ageing [19]. Age-related behavioral changes similar to humans were found in several animal studies, reporting increased depression- [20, 21], anhedonia- [22, 23], anxiety-like behavior [24] and also cognitive impairment [25]. Experimental studies dealing with behavioral sex differences in aging are lacking.

An animal model of androgen deficiency is the removal of the primary endogenous androgen source – the testes – via gonadectomy (GDX). Gonadectomized animals subsequently display increased anxiety- [26, 27] and depression-like [28] behavior, impaired cognitive performance [29–31] and altered social behavior [32]. It was shown that these negative effects of GDX can be reversed by administration of testosterone [26–28, 30], although some studies reported different and conflicting results [33, 34].

It is not clear, whether the length of androgen deficiency could affect the behavioral outcomes and lead to inconsistencies of the published data. The vast majority of studies are focusing on the effect of androgen deficiency on behavior of tested animals that were androgen-deficient only for a short time (weeks to months). The clinical relevance of such short-term effect is questionable.

The aim of our study was to describe sex differences in behavior and long-term effects of androgen deficiency in aged rats. In addition, the acute effect of a single testosterone injection on depression-like behavior was observed.

## RESULTS

### Experiment 1: Behavioral phenotyping of aged Wistar rats

#### Locomotor activity

**Figure 1:**
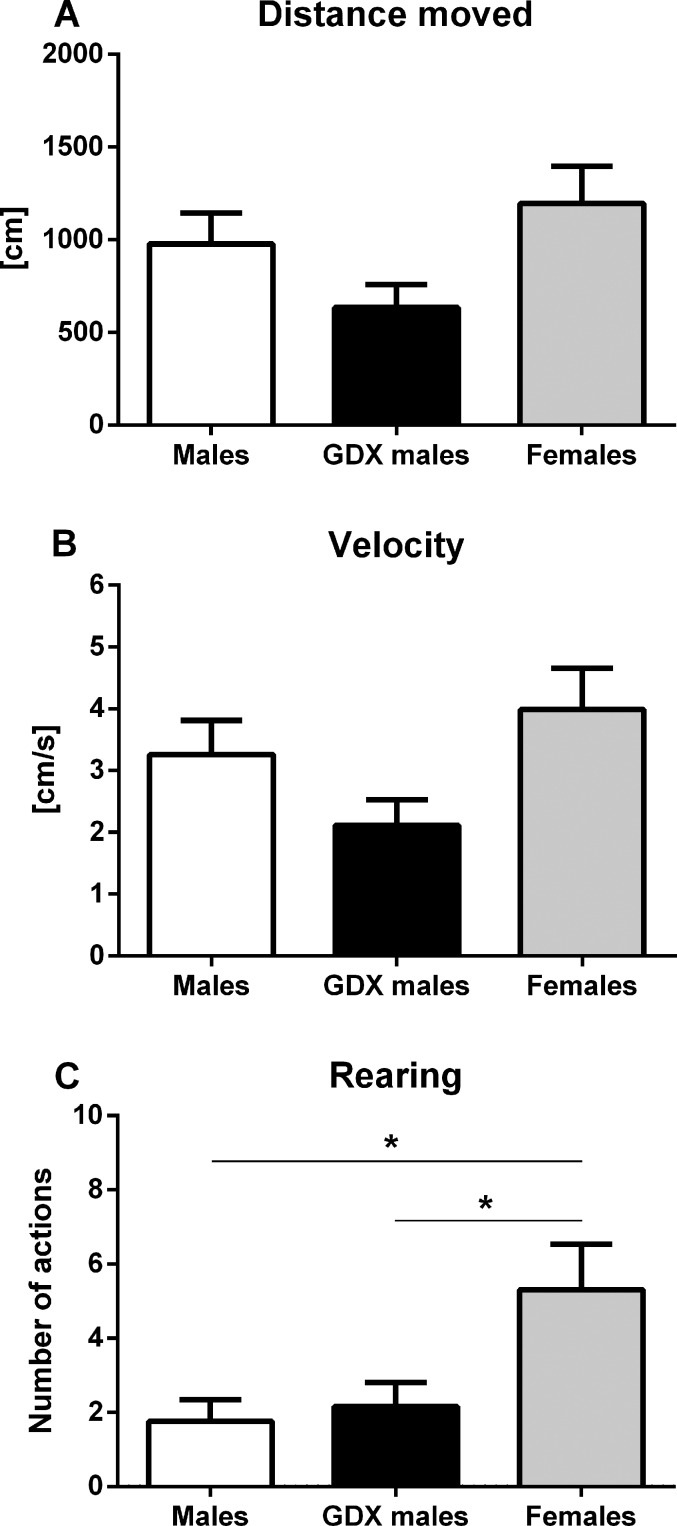
Locomotor activity of aged rats in the open field test (**A, B**) Horizontal and (**C**) vertical locomotor activity for males (white bar), GDX males (black bar) and females (grey bar). There were no significant differences between groups in horizontal locomotor activity. Females had a significantly higher vertical locomotor activity compared to both male groups (p<0.05). Data are expressed as means + SEM. *p<0.05.

In the open field, no significant differences were found in total distance moved (horizontal locomotor activity) (F=2.876, p=0.068, Fig. 1A) and in average velocity of the movement (F=2.876, p=0.068, Fig. 1B). Significant differences were found in the number of rears (vertical locomotor activity), where females displayed significantly higher rearing frequencies (F=4.889, p<0.05, Fig. 1C) compared to male groups.

#### Anxiety-like behavior

**Figure 2:**
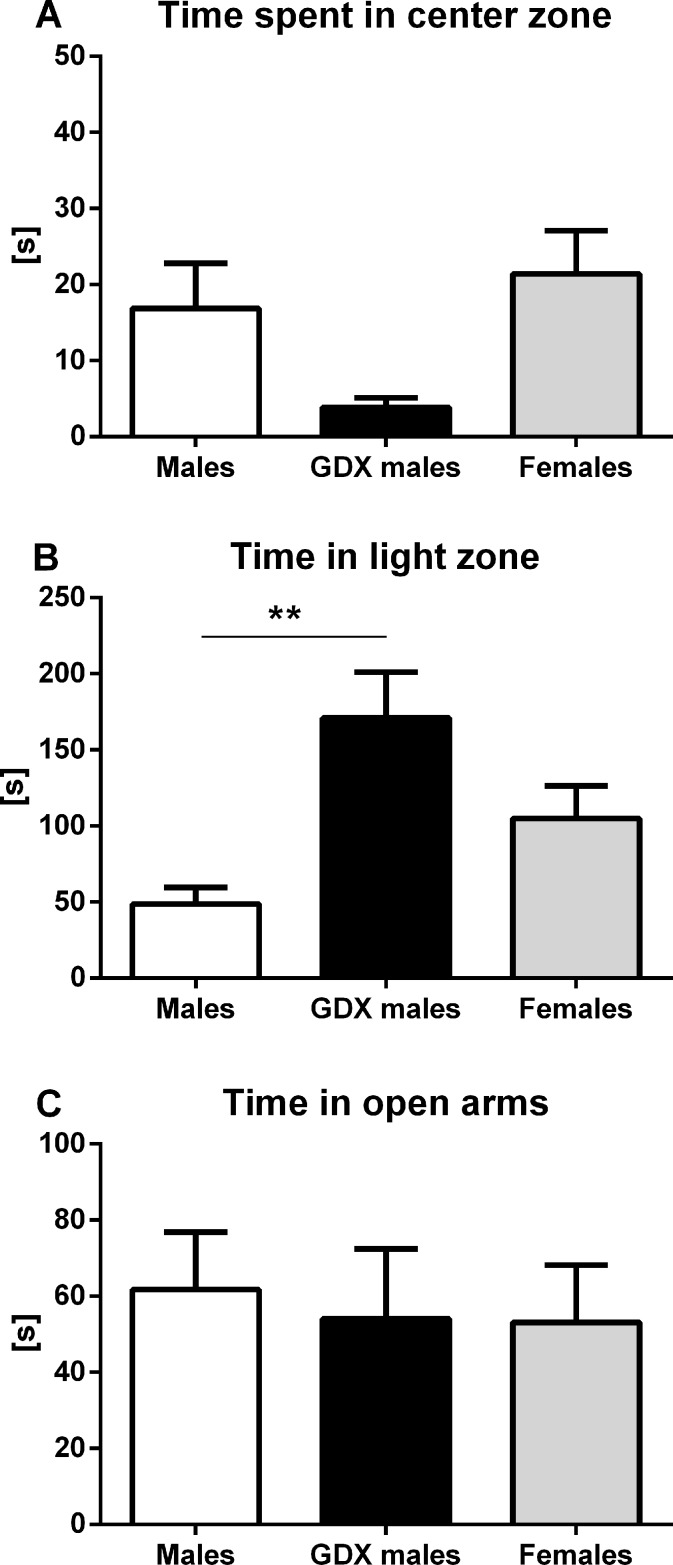
Anxiety-like behavior in aged rats (**A**) Time spent in the center zone of the open field, (**B**) time spent in the light zone of the light/dark box and (**C**) time spent in the open arms of the elevated plus maze. No significant difference between the groups was found in the time spent in the center zone. GDX males were less anxious than males in light/dark box (p<0.01). There was no significant difference in the time spent in open arms between groups in elevated plus maze. Males (white bar), GDX males (black bar) and females (grey bar). Values are expressed as means + SEM. **p<0.01.

Anxiety-like behavior was assessed in three types of behavioral tests. In open field, no significant differences were detected in time spent in the center zone, although GDX males tended to spend less time in center zone compared to females (F=2.940, p=0.067, Fig. 2A). In light/dark box, GDX males spent longer time in the light compartment than control males (F=7.656, p<0.01, Fig. 2B). The elevated plus maze test did not show any significant differences in time spent in open arms between groups (F=0.085, p=0.919, Fig. 2C).

#### Exploratory activity and memory

**Figure 3:**
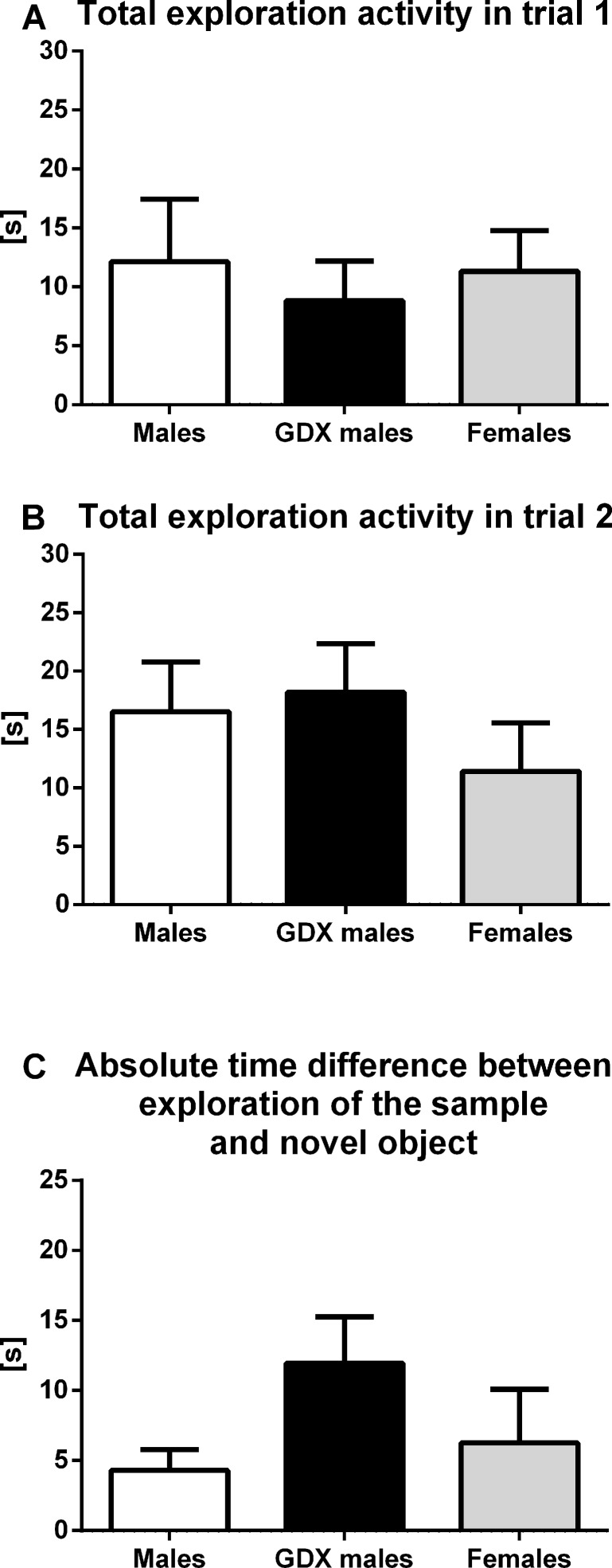
Exploratory activity and memory in aged rats (**A**) Total time spent interacting with each individual object in trial 1 and (**B**) in trial 2. (**C**) Absolute time difference between investigating the sample and novel object. There were no significant differences in any of observed parameters between groups. Males (white bar), GDX males (black bar) and females (grey bar). Values are expressed as means + SEM.

No group differences were observed in overall exploratory activity (total time spent interacting with each individual object) for either trial 1 (F=0.170, p=0.844, Fig. 3A) or trial 2 (F=0.673, p=0.516, Fig. 3B) in novel object recognition task. Furthermore, the absolute time difference between investigating the sample and novel object did not show any significant difference among groups (F=1.845, p=0.172, Fig. 3C).

#### Social interaction test

**Figure 4:**
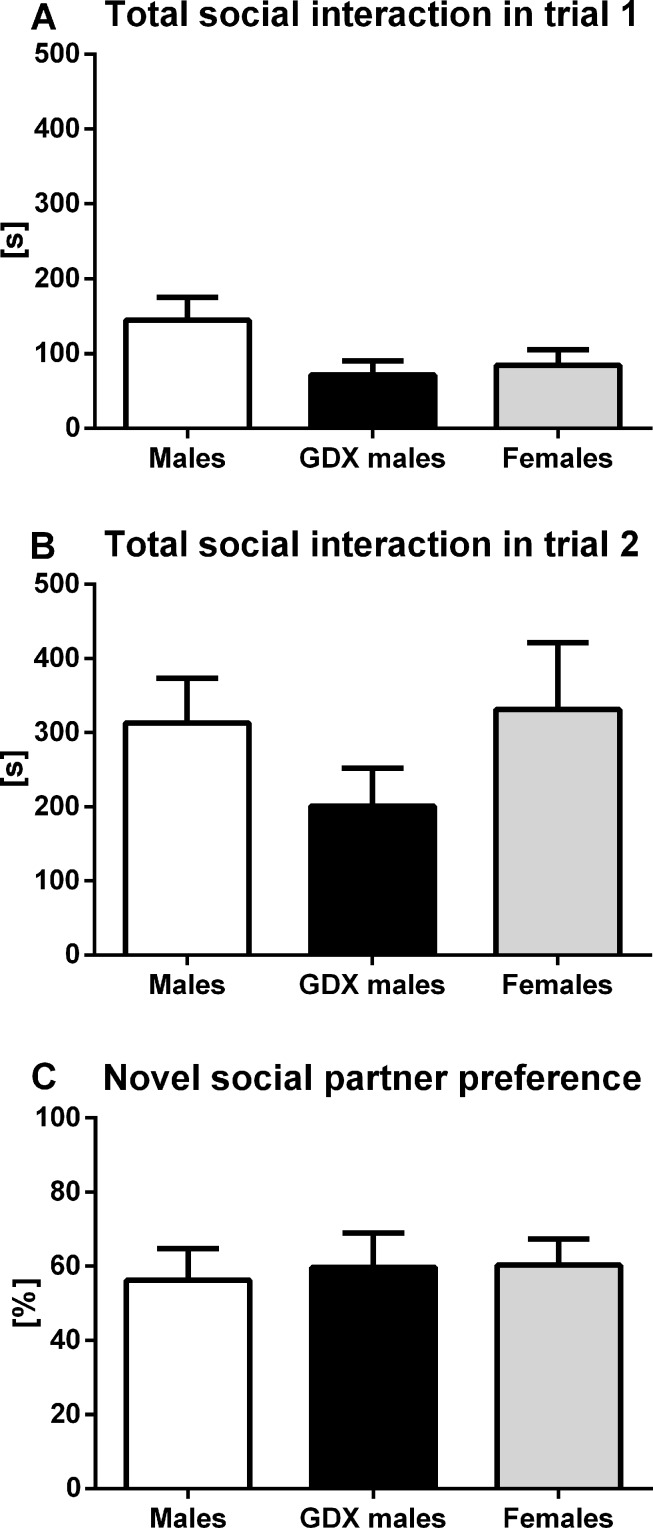
Sociability and preference for social novelty in aged rats (**A**) Total social interaction time in trial 1 and (**B**) trial 2. (**C**) Preference for novel social partner in trial 2 for males (white bar), GDX males (black bar) and females (grey bar). There were no statistically significant differences in the analyzed parameters between groups. Data are expressed as means + SEM.

There were no significant differences between groups of aged rats in total social interaction time in either trial 1 (F=2.53, p=0.094, Fig. 4A) or trial 2 (F=1.11, p=0.340, Fig. 4B). Similarly, no significant difference was observed in the preference for novel social partner between groups (F=0.072, p=0.930, Fig. 4C).

.

#### Forced swim test

**Figure 5:**
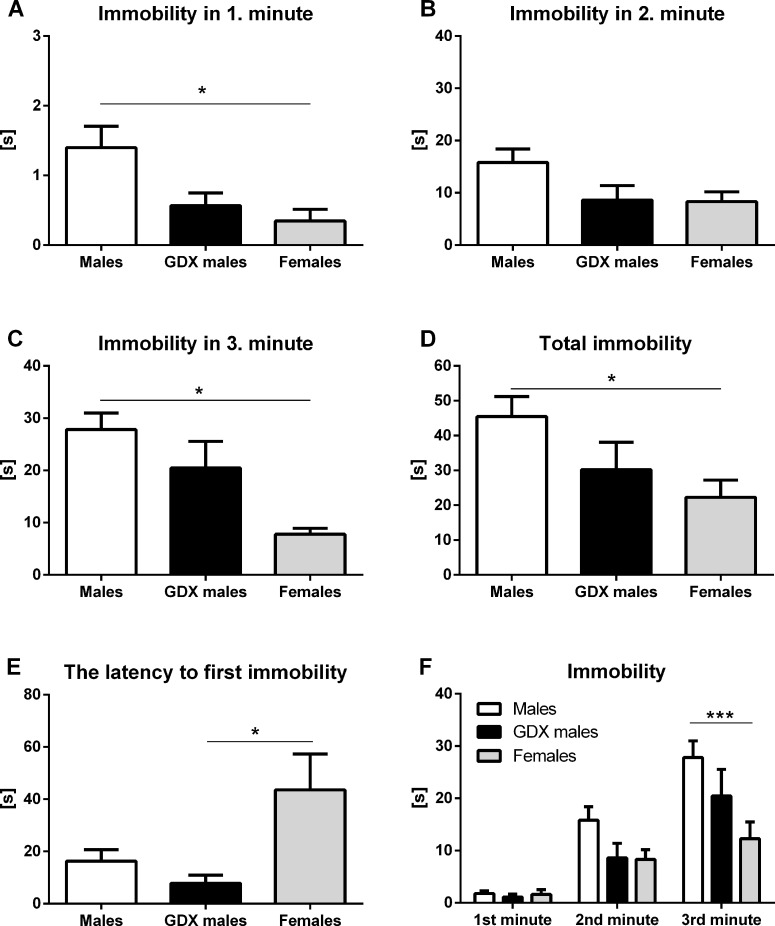
Immobility duration in the forced swim test Immobility time in the (**A**) 1^st^ min, (**B**) 2^nd^ min, (**C**) 3^rd^ min, (**D**) during the total of 3 min testing period, (**E**) the latency to first immobility and (**F**) duration of the immobility taken minute by minute over the 3 min testing period. Males had longer immobility duration in the 1^st^ min and 3^rd^ min compared to females (p<0.05). This difference was also observed in total immobility time during the 3 min testing period (p<0.05). GDX males displayed a shorter latency to first immobility compared to females (p<0.05). Males (white bar), GDX males (black bar) and females (grey bar). Data are expressed as means + SEM. *p<0.05, ***p<0.001.

In forced swim test, male rats spent significantly more time immobile in the 1st min (F=5.45, p<0.01, Fig. 5A) and 3rd min (F=6.50, p<0.01, Fig. 5C) compared to females. There were no significant differences in the 2nd min between groups (F=3.04, p=0.061, Fig. 5B). Im-mobility time recorded during the total of 3 min testing period was significantly increased in males compared to females (F=3.48, p<0.05, Fig. 5D). A significant decrease in latency to first immobility was detected in GDX males compared to females (F=4.707, p<0.05, Fig. 5E). Two-way ANOVA repeated measures revealed significant effect of time (F=60.9, p<0.001) and treatment (F=3.481, p<0.05) on immobility duration in phenotyping session. There was also a significant interaction between time and treatment (F=3.71, p<0.01). Minute-by-minute analysis revealed gender differences in the 3rd min of the forced swim test, where control males spent significantly more time immobile compared to females (p<0.001, Fig. 5F).

#### Anhedonia-like behavior

**Figure 6:**
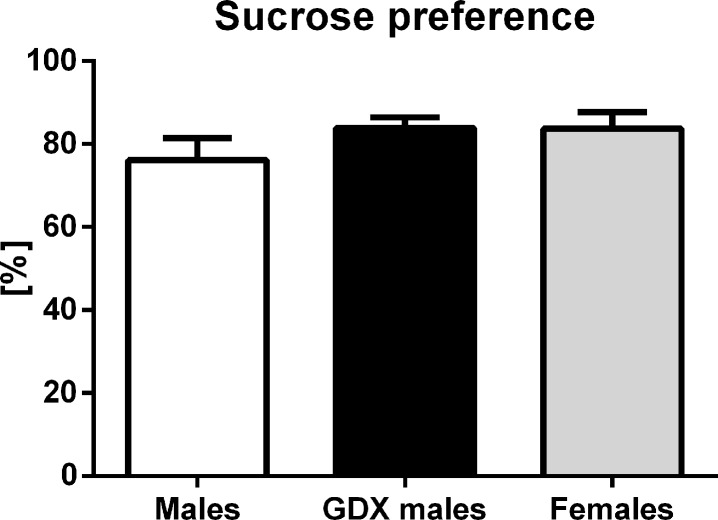
Sucrose preference test Percentual preference for 2% sucrose solution of total 24h liquid intake. No differences in the preference for sucrose between the groups was detected. Males (white bar), GDX males (black bar) and females (grey bar). Values are expressed as means + SEM.

There were no statistically significant differences in the preference for sucrose-sweetened water between groups (F=1.10, p=0.343, Fig. 6).

#### Instrumented observation cage

**Figure 7:**
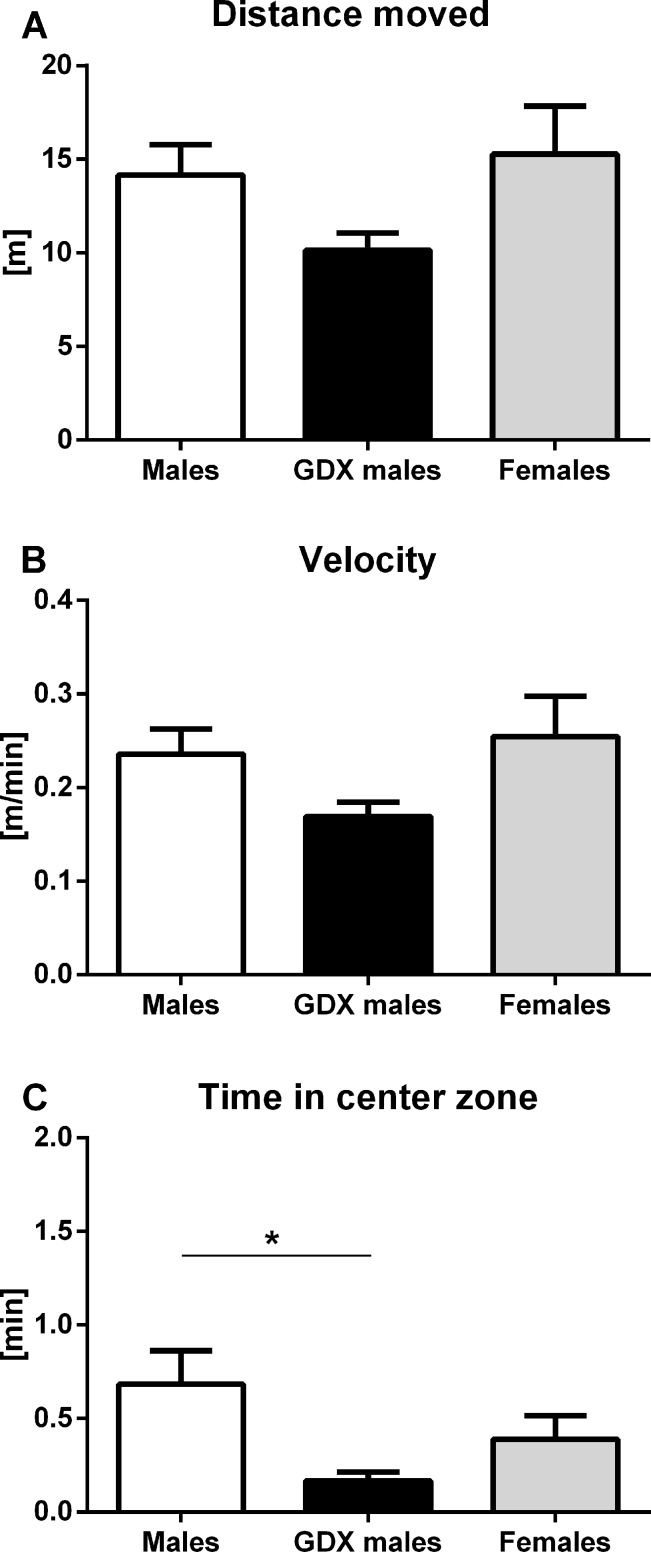
Locomotor activity and anxiety-like behavior of aged rats in instrumented observation cage (**A, B**) No significant differences between the groups were found in locomotor activity. (**C**) GDX males spent significantly less time in the center zone than males. Males (white bar), GDX males (black bar) and females (grey bar). Values are expressed as means + SEM. *p<0.05.

No differences were observed in horizontal locomotor activity measured by distance moved and average velo-city of the movement during the total of 1 h testing period (F=2.354, p=0.109) between groups (Fig. 7A, B). GDX males spent significantly less time in the center zone compared to intact males (F=3.774, p<0.05, Fig. 7C).

#### Plasma testosterone concentration

**Figure 8:**
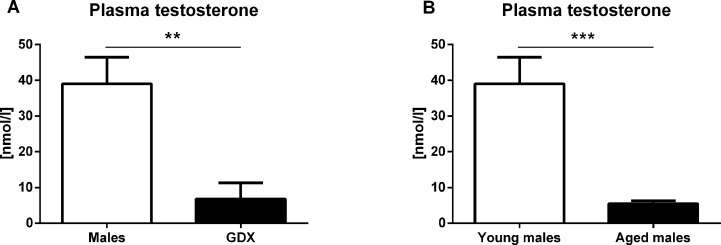
Plasma testosterone concentration (**A**) Plasma testosterone concentration 4 weeks following gonadectomy. Gonadectomy led to a significantly lower testosterone concentration compared to control males (p<0.01). Males (white bar) and GDX males (black bar). (**B**) Plasma testosterone concentration in young and aged male rats. Aged males (30 months) had significantly lower plasma testosterone concentration compared to young males (p<0.001) [young males (white bar), aged males (black bar)]. Values are expressed as means + SEM. **p<0.01, ***p<0.001.

In young adults, significantly lower testosterone concentration was detected four weeks following gonadectomy (F=2.775, p<0.01, Fig. 8A). Significantly lower plasma testosterone concentration was also detected in aged male rats compared to young males (F=63.30, p<0.001, Fig. 8B).

### Experiment 2: The acute effect of testosterone on depression-like behavior in aged Wistar rats

**Figure 9:**
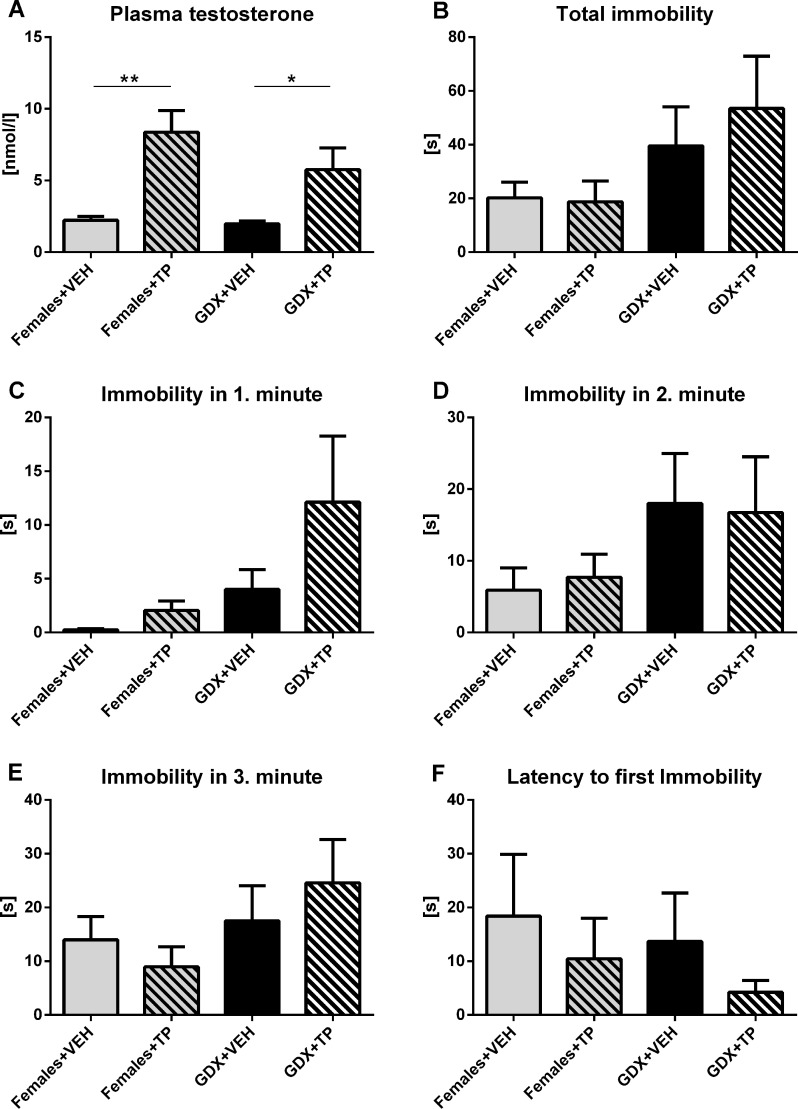
Immobility duration in forced swim test after acute testosterone treatment **(A)** Plasma testosterone concentration 30 minutes after acute treatment with olive oil vehicle (VEH) or testosterone propionate (TP) in aged GDX males and aged females. Acute testosterone treatment caused an increase in plasma testosterone concentration in both GDX males and females compared to their vehicle treated controls (p<0.01). (**B**) Immobility time in the forced swim test during the 3 min testing period, (**C**) immobility duration in the 1^st^ min, (**D**) 2^nd^ min, (**E**) 3^rd^ min and (**F**) the latency to first immobility without any significant differences in observed behavioral parameters between groups. Values are expressed as means + SEM. *p<0.05, **p<0.01. Females+VEH (grey bar), Females+TP (grey striped bar), GDX+VEH males (black bar), GDX+TP males (black striped bar).

To examine the acute effect of testosterone on depressive-like behavior, GDX males and females received subcutaneous injection of testosterone propionate (TP) or vehicle (olive oil) 20 min before testing in forced swim test. Acute testosterone treatment caused an increase in plasma testosterone concentration in both GDX males and females compared to their vehicle treated controls (p<0.01, Fig. 9A). There were no significant differences in any of observed parameters of the forced swim task including total immobility duration (p=0.409, Fig. 9B), time spent immobile in the 1^st^ min (p=0.088, Fig. 9C), in 2^nd^ min (p=0.52, Fig. 9D), in 3^rd^ min (p=0.394, Fig. 9E) and in the latency to first immobility (p=0.767, Fig. 9F) between groups.

## DISCUSSION

In the current experiment, the effect of long-term androgen deficiency on behavior of aged male rats was analyzed. Although GDX male rats had lower plasma testosterone concentration than control male rats, gonadectomy did not lead to any behavioral differences described in young GDX rats. No differences were found between GDX males and controls in locomotor activity, exploratory activity and memory, social interaction, hedonic or depression-like behavior. Regarding anxiety-like behavior, long-term androgen depletion had anxiogenic and anxiolytic effect in a test dependent manner. In the past, the differences between tests used for characterization of anxiety have been described [35]. But the conclusive recommendation was to use several tests to robustly evaluate anxiety [36]. We have followed that recommendation and conducted the open field, elevated plus maze and the light-dark box. The results were inconclusive, partially even directly opposite to each other. We only can conclude that no consistent effect of gonadectomy on anxiety was found in the aged male rats. This comprehensive behavioral phenotyping of aged rats revealed no major role of endogenous testosterone.

The only consistent significant sex difference was observed in depression-like behavior, where males spent longer time immobile than females. This is in line with the findings of a previous study analyzing the behavior of aged mice [20]. However, in that study single administration of testosterone or other androgens had an antidepressive effect. In our study, acute testosterone treatment had no effect on depression-like behavior of aged rats. The main difference between the studies besides the species used (mice vs. rats) is the gonadal status of the treated male animals – intact vs GDX. It is, however, unclear whether this fact can explain the differences in the outcomes. Especially, as some studies on young rats showed no effect of acute testosterone administration on anxiety- and depression-like behavior as well [33].

Age-related decline in androgen concentration of male rats is detectable around 13 months of age and further decrease is observed at age of around 24 months as described decades ago [37]. In our study, we have confirmed that male rats at the age of 30 months have much lower testosterone concentrations than young adults – cca. 15%. Gonadectomy, nevertheless, decreased testosterone even further to 5% in comparison to young adult male rats. However, the aim of our study was not to observe the age-related cognitive decline or behavioral changes associated with aging, but rather to analyze sex differences and the role of endogenous testosterone in aging. Although numerous studies showed that hypogonadism in aged men result in depression and anxiety [16–18] and that supplementation with testosterone has beneficial effects [16, 18, 38], relevant experimental studies on rodents analyzing the behavior in detail are scarce. The cause might likely be the issues and costs related to long-term housing of animals after gonadectomy – more than two years in this experiment.

This experiment is unique as it analyzes long-term effects of gonadectomy and, thus, the role of endogenous testosterone on a number of behavioral outcomes in aged rats. But it is also affected by several limitations. We have not analyzed the dynamic changes during aging. Differences that we have not observed might have been present at the age of 10 or 20 months. However, to prevent bias from repeating the behavioral tests [39] we decided to conduct behavioral phenotyping only at the end of the experiment.

Although the rats were apparently healthy at the time of testing, we have not screened the animals for age-related metabolic diseases that might increase the variability in the observed behavioral measures. The outcome in the interventional experiment might be positive if long-term testosterone administration would be chosen. Based on published interventional experiments with single testosterone injection in younger animals antidepressive effects of the treatment could be expected. The lack of effects might be caused by the altered pharmacokinetic profile of testosterone [40] or by the complex metabolism of testosterone in the brain. In particular, aromatase converting testosterone to estradiol, but also androgen and estrogen receptors are differentially expressed in some regions of the aging rat brain as recently described [41]. This underscores the importance of conducting experiments focusing on the effects of exogenous testosterone on brain functions in animals of various age including very old rats as in this experiments.

In conclusion, the findings of the present experiment did not confirm the effect of either aging or gonadectomy on behavior of rats in the variety of behavioral tasks. Sex differences in behavior were found only in forced swim test, where males displayed longer immobility time than females. Additionally, acute androgen replacement failed to change behavior of aged rats. Thus, the outcomes of the present study do not support testosterone as an anti-aging agent. Further more detailed and focused studies are, however, needed for the interpretation of our results regarding their relevance for the postulated hyperfunction theory of aging [42, 43].

In summary, comprehensive behavioral phenotyping of aged gonadectomized rats did not reveal a major role of endogenous testosterone as factor determining the behavior in aged male rats. Sex differences in aged animals were found only in depression-like behavior with male sex being the risk factor. In the interventional experiment no effect of exogenous testosterone on depression-like behavior was observed. Whether the reported findings have any relevance for androgen replacement therapy in hypogonadism needs further elucidation.

## MATERIALS AND METHODS

### Ethics statement

All procedures have been conducted in accordance with Slovak legislation and were approved by the ethical committee of the Institute of Molecular Biomedicine, Comenius University, Bratislava.

### Animals and housing conditions

In this study, Wistar albino rats (n = 42) were used. Young animals were purchased from the breeding colony Anlab (Prague, Czech Republic). The rats were group-housed (4-6 per cage) throughout the study in polycarbonate cages (50 × 36 × 19cm). All rats were kept under controlled laboratory conditions (temperature 22 ± 2°C, humidity 55 ± 10%) with a maintained 12:12 light-dark cycle. Rats had *ad libitum* access to food and water.

### Surgery

Rats were randomly divided into 3 groups: control males (n=15), gonadectomized males (GDX, n =14), and females (n=13). Gonadectomy was performed on postnatal day 47. Rats were anaesthetized with intraperitoneal injection of ketamine (100 mg/kg, Narkamon inj, Bioveta, Czech Republic) and xylazine (10 mg/kg, Xylariem inj, Riemser, Germany). In gonadectomized rats, both testes (with cauda and caput epididymis) were extracted through a small incision made at the posterior tip of the scrotum. The vas deferens and spermatic blood vessels were ligated with a silk suture. After surgery, rats were group-housed with their previous cage mates and had free access to water and food until the age of 30 months.

### Experiment 1: Behavioral phenotyping of aged Wistar rats

At the age of 30 months, rats underwent behavioral phenotyping using a battery of tests available at our institute. These include assessment of locomotor activity and anxiety-like behavior (open field, elevated plus maze and light-dark transition task), memory functions (novel object recognition test), sociability (social interaction test) and depression-like behavior (sucrose consumption test and forced swim test). The method of each behavioral test is described below. Except the sucrose consumption test all other tests were recorded in a slightly lit room using automated tracking software EthoVision XT 10.1 (Noldus, Wageningen, The Netherlands). All behavioral tests were carried out during the light phase of the light/dark cycle.

### Open field test

The open field apparatus consisted of a square arena (100cm x 100cm) virtually divided into the central zone (40cm x 40cm) and border zone. Testing of animals was carried out between 11:00 - 12:00. Rats were placed into the center of the arena and were allowed to freely explore it for 5 min. Total distance moved and average velocity of the movement were monitored to evaluate locomotor activity. The time spent in the center zone was considered as anti-anxiety activity [44]. The open field arena was cleaned with a damp cloth containing Incidur spray (Ecolab, Dusseldorf, Germany) between the trials.

### Elevated plus maze

The elevated plus maze apparatus (elevated to a height of 50cm above the floor) consisted of two open (45cm x 10cm) and two closed arms (45 × 10 × 40cm) extending from a central platform (10cm x 10 cm). Testing of animals was conducted between 12:00 - 13:00. Rats were placed into the central zone facing the open arm and were allowed to freely explore the maze for 5 min. The time spent in closed arms was considered to be an indicator of anxiety [44]. The arena was cleaned with a damp cloth containing Incidur spray (Ecolab, Dusseldorf, Germany) between trials.

### Light-dark transition task

The rectangular light/dark box (65 × 45 × 30cm) consisted of equally sized dark (closed with a lid) and light (brightly illuminated) parts. Testing of animals was performed between 13:00 - 14:00. Animals were placed into the light compartment and were allowed to freely explore both compartments for 5 min. The time spent in the dark part of the box indicated anxiety [45]. The light/dark box was cleaned with a damp cloth containing Incidur spray (Ecolab, Dusseldorf, Germany) between trials.

### Novel object recognition

The novel object recognition testing procedure was a slightly adapted version of the object recognition test described by Havranek [46]. The test was performed in the open field arena (100cm x 100cm) between 11:00 - 13:00. The open field test was carried out one day before testing and was considered as a habituation trial. The test consisted of two trials – trial 1 and trial 2, 5 min each, separated by a retention interval of 1 hour. Three different objects were used in the task: 1. a standard 750mL plastic bottle, 2. a 205mL metallic can and 3. 1L glass bottle. All objects were filled with water. In trial 1, the animals were placed in the middle of arena with two objects (plastic and metallic bottle) situated at the opposite corners of the arena (27cm from the wall and 55cm apart of each other). Time spent exploring the object 1 or 2 was marked as “a1” or “a2”. In trial 2, the plastic bottle was left in the arena (time “a3”) and the metallic can was replaced with a novel object (glass bottle, time “b”). During both trials, the animals could freely explore the arena and objects. The apparatus, as well as the objects, were cleaned after each animal with Incidur spray (Ecolab, Dusseldorf, Germany) to prevent any olfactory disturbance. Recorded parameters were as follows: total time exploring both objects in trial 1 and trial 2 (trial 1: e1 = a1 + a2, trial 2: e2 = a3 + b), the absolute time difference between investigating the sample and the novel object (trial 2: d1 = b - a3). Calculation of these parameters was based on a previously published protocol [47].

### Social interaction test

In the social interaction test, the open field arena was used as testing apparatus. Testing of animals was done between 15:00 - 18:00. The test consisted of two subsequent trials (trial 1 and trial 2) of 10 min duration each. In trial 1, tested animal as well as a social partner (stranger 1) were placed in opposite corners of the open field arena and allowed to socially interact for 10 min. After completion of the first trial, another social partner (stranger 2) was placed into the arena for second 10-min trial. In trial 1, the stranger 1 served as a social partner allowing observation of social ability of tested animals. In trial 2, stranger 1 served as a familiar social partner and stranger 2 as a novel social partner allowing evaluation of social novelty preference of tested animals. In both trials, the following behavioral parameters initiated by the tested animal toward the stranger 1 or stranger 2 were observed: sniffing, following, aggressive behavior and comfort behavior in close distance to social partner. These individual parameters were summed up as the overall social activity of the tested animals and subsequently evaluated for the trial 1 (interaction with the stranger 1) and for the trial 2 (interaction with familiar stranger 1 and novel stranger 2). Novel social partner preference was calculated as total interaction time with stranger 2/ total interaction time with stranger 1 + stranger 2. The arena was cleaned with a damp cloth containing Incidur spray (Ecolab, Dusseldorf, Germany) between trials.

### Forced swim test

In forced swim test, rats were individually placed into a transparent plastic cylinder (height 45 cm, diameter 30 cm) filled with tap water (25 ± 1°C). The time of immobility (with immobility threshold 5%) was recorded during 3 min as an index of depression-like behavior. The higher immobility time indicates depression-like behavior [48]. Immobility was defined as minimal movements required for keeping the head above water. The immobility time was assessed in minute-by-minute time bins. The total immobility time and the latency to the first immobility were also analyzed during the 3 min testing period. Animals were dried with towels before returning to their home cage. The testing took place between 15:00-18:00.

### Sucrose preference test

The sucrose preference test consisted of a two-bottle choice paradigm. Rats were placed in metabolic cages for 24 hours. During this period, rats were given access to two bottles, one with 2% sucrose solution and a second with tap water. To eliminate side preference artifact, the position of the bottles was switched after 12 hours. The rats had no access to food with food deprivation during the entire length of the test. No previous food or water deprivation was applied prior the test. Sucrose preference was tested without prior exposure to sucrose. The consumption of water and sucrose solution was measured by weighing the bottles and subtracting the volume of the spilled out liquid in a special container of metabolic cages at the end of the test. The preference for sucrose was calculated as the percentage of the consumed sucrose solution from the total volume of liquid drunk. The decreased preference for sucrose was considered as a mark of anhedonia-like behavior [49, 50].

### Instrumented observation cage – PhenoTyper

The PhenoTyper cage consists of a bottom plate (45cm x 45cm), four replaceable walls with ventilation holes and a PhenoTyper top unit including a videorecorder. It allows uninterrupted behavioral testing for a longer time (Noldus, Wageningen, The Netherlands). To examine the locomotor activity for a longer period, aged rats were placed into PhenoTyper cages for 60 min. The measured behavioral parameters were as follows: total distance moved, average velocity (locomotor activity) and time spent in the center zone (anti-anxiety beha-vior). The testing was performed between 12:00 - 15:00.

### Experiment 2: The acute effect of testosterone on depression-like behavior in aged Wistar rats

To examine the acute effect of testosterone on depression-like behavior the previously described forced swim test was conducted again. GDX males and intact females received subcutaneous injection of 1 mg/kg testosterone propionate (TP; in olive oil, Sigma-Aldrich Chemie GmbH, Germany) (GDX+TP, n=6; Females+TP, n=6) or olive oil vehicle (GDX+VEH, n=5; Females+VEH, n=5) 20 min before forced swim test. It has previously been demonstrated, that this testosterone dose results in physiological concentrations of testosterone in intact aged rats [34], young GDX rats [51, 52] and mice [53]. The experiment was performed between 15:00 - 18:00.

### Blood collection and hormone assays

To assess plasma testosterone concentrations, blood samples (approximately 300 μl) were collected from the tail vein. Blood samples were centrifuged at 2000g for 5 min. Plasma samples were stored at –20°C until further analyses. Concentration of testosterone in plasma was measured using the commercially available ELISA kit (DRG Diagnostic, Marburg, Germany). The antibody cross-reacts 100% with testosterone, and shows minor cross-reactivity with 5α-dihydrotestosterone (0.8%), androstenedione (0.9%), 11β-hydroxyestosterone (3.3%), 17α-methyltestosterone (0.1%), 19-nortes-tosterone (3.3%), epitestosterone (<0.1%), estradiol (<0.1%), progesterone (<0.1%), cortisol (<0.1%), estrone (<0.1%), danazol (<0.1%). The analytical sensitivity was 0.083 ng/ml. The average intra-assay coefficient of variation for the testosterone assay was below 5% and inter-assay coefficient of variation was below 10%.

The first measurement of plasma testosterone was performed four weeks after gonadectomy to verify the effectiveness of the surgery. The next testosterone assessments were done at the age of 30 months and 30 minutes after acute testosterone treatment.

### Statistical analysis

Statistical analysis was performed using the GraphPad Prism version 6.01 (GraphPad Software, Inc., CA, USA). In experiment 1, to analyze behavior of aged rats during phenotyping, one-way analysis of variance (ANOVA) with Bonferroni-corrected post-hoc t-test was used. Statistical comparison of plasma testosterone concentration between young and aged rats and also between control and gonadectomized rats was performed using unpaired students' two-tailed t-test. In experiment 2, to evaluate behavior of aged rats after acute testosterone treatment, the non-parametric Kruskal-Wallis test with Dunn's multiple comparison test was used. Where appropriate time series were analyzed using two-way repeated measures ANOVA (independent factors: treatment and time). P values lower than 0.05 were considered statistically significant. Data are presented as mean plus standard error of the mean (SEM).
